# Circulating dipeptidyl peptidase 3 and outcomes in acute heart failure: an analysis of the STRONG-HF and CORTAHF studies

**DOI:** 10.1093/eschf/xvag076

**Published:** 2026-03-16

**Authors:** Jolie Bruno, Christopher Edwards, Koji Takaji, Feriel Azibani, Beth Davison, Gad Cotter, Karine Santos, Oliver Hartmann, Andrew P Ambrosy, Alexandre Mebazaa, Adrien Picod

**Affiliations:** INSERM UMR-S 942, Cardiovascular Markers in Stress Condition (MASCOT), 2 rue Ambroise Paré, Paris 75010, France; Université de Paris Cité, 45 Rue des Saints-Pères, Paris 75006, France; Department of Cardiology, Inselspital, Bern University Hospital, University of Bern, Freiburgstrasse 20, 3010 Bern, Switzerland; Momentum Research, Inc., 3100 Tower Blvd # 802, Durham, NC 27707, USA; Momentum Research, Inc., 3100 Tower Blvd # 802, Durham, NC 27707, USA; INSERM UMR-S 942, Cardiovascular Markers in Stress Condition (MASCOT), 2 rue Ambroise Paré, Paris 75010, France; Momentum Research, Inc., 3100 Tower Blvd # 802, Durham, NC 27707, USA; Momentum Research, Inc., 3100 Tower Blvd # 802, Durham, NC 27707, USA; 4TEEN4 Pharmaceuticals GmbH, Neuendorfstraße 15A, 16761 Hennigsdorf, Germany; 4TEEN4 Pharmaceuticals GmbH, Neuendorfstraße 15A, 16761 Hennigsdorf, Germany; Department of Cardiology, Kaiser Permanente San Francisco Medical Center, 2238 Geary Blvd #8, San Francisco, CA 94115, USA; Division of Research, Kaiser Permanente Northern California, Pleasanton, CA, USA; INSERM UMR-S 942, Cardiovascular Markers in Stress Condition (MASCOT), 2 rue Ambroise Paré, Paris 75010, France; Université de Paris Cité, 45 Rue des Saints-Pères, Paris 75006, France; Department of Anesthesiology, Critical Care and Burn Unit, University Hospitals Saint-Louis et Lariboisière, AP-HP, Paris, France; INSERM UMR-S 942, Cardiovascular Markers in Stress Condition (MASCOT), 2 rue Ambroise Paré, Paris 75010, France; Medical and Surgical Intensive Care Unit, Hôpital Avicenne, AP-HP, Bobigny, France; Université Sorbonne Paris Nord, 99 Av. Jean Baptiste Clément, 93430 Villetaneuse, France

**Keywords:** Dipeptidyl peptidase 3, Acute heart failure, Outcomes, Guideline-directed medical therapy

## Abstract

**Introduction:**

Circulating dipeptidyl peptidase 3 (cDPP3) is implicated in cardiocirculatory failure and elevated concentrations predict poor outcomes in shock states. Its role in acute heart failure (AHF) is unexplored. We assessed the clinical relevance of cDPP3 in AHF.

**Methods:**

Analyses were performed using two prospective AHF trials: STRONG-HF and CORTAHF. cDPP3 was measured at baseline and follow-up (day 90 in STRONG-HF; day 30 in CORTAHF). Associations with 180-day (STRONG-HF) and 90-day (CORTAHF) outcomes were evaluated according to baseline concentrations. Longitudinal changes during guideline-directed medical therapy (GDMT) optimization and predictors of elevated cDPP3 were analysed.

**Results:**

In STRONG-HF, 222/973 patients (23%) had cDPP3 ≥ 40 ng/mL. These patients were younger (58 ± 15 vs. 65 ± 13 years, *P* < .0001), more frequently female (47.7% vs. 35.8%, *P* = .0013) and Black (42.8% vs. 14.4%, *P* < .0001), with lower NT-proBNP concentrations (*P* < .0001). Baseline cDPP3 ≥ 40 ng/mL was not associated with 180-day outcomes. Over 90 days, cDPP3 decreased by −15% with high-intensity care and −8% with usual care (*P* = .078). Changes in cDPP3 were not associated with NT-proBNP reduction (continuous *P* = .797; ≥30% responder *P* = .990). In pooled multivariable analysis, MRA use was independently associated with cDPP3 ≥ 40 ng/mL (OR 3.83; 95% CI 1.47–9.96; *P* = .006), whereas non-Black ethnicity was associated with lower odds (OR 0.43; 95% CI 0.29–0.64; *P* < .0001).

**Conclusion:**

In AHF, cDPP3 was mildly elevated and was not associated with clinical outcomes or congestion relief during GDMT optimization. Elevated cDPP3 identified a distinct clinical phenotype but did not confer adverse prognosis.

## Graphical Abstract

**Graphical Abstract:**
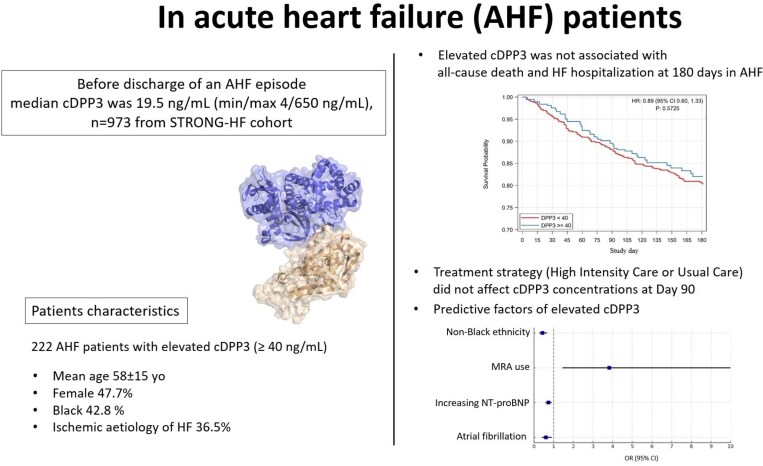
cDPP3 concentrations in acute heart failure, showing mildly elevated values compared to the reference range, no relation to outcomes or treatment strategy, and identifiable clinical predictors of increased levels. Abbreviations: AHF, acute heart failure; cDPP3, circulating dipeptidyl peptidase 3; HF, heart failure; MRA, mineralocorticoid receptor antagonist; NT-proBNP, N-terminal pro–B-type natriuretic peptide.

## Introduction

Circulating dipeptidyl peptidase 3 (cDPP3) is an emerging cardiovascular biomarker, zinc-dependent metallopeptidase that degrades bioactive peptides, including key substrates such as angiotensin II, thereby modulating haemodynamic and inflammatory pathways.^1^ By inactivating angiotensin II, cDPP3 may contribute to dysregulation of the renin–angiotensin–aldosterone system (RAAS) and impaired angiotensin II type 1 receptor (AT1R) signalling, leading to systemic vasodilation and hypotension,^1^ key features of cardiocirculatory failure.

Recent studies have heightened interest in cDPP3, demonstrating that elevated concentrations, which directly reflect increased enzymatic activity, are associated with worse outcomes in critically ill patients, including refractory shock, higher vasoactive support, and death.^2–7^ These associations become significant at concentrations above 40 ng/mL, corresponding to the 97.5th percentile of the physiological range; in contrast, median cDPP3 concentrations in healthy individuals are around 10–15 ng/mL.^8–10^ In addition, an early decline in cDPP3 within the first few days is associated with a lower risk of adverse outcomes.^2–6,9^ However, subgroup analyses suggest that the prognostic value of baseline cDPP3 may vary depending on the underlying cause of cardiovascular failure, being more pronounced in non–acute myocardial infarction shock.^9^

Preclinical studies have demonstrated that elevated cDPP3 can affect vascular tone, myocardial contractility, and renal haemodynamics, while targeted inhibition with the monoclonal antibody procizumab improves haemodynamic stability and organ perfusion.^11–13^ These findings support the therapeutic potential of cDPP3 inhibition, and its clinical efficacy in humans is currently being investigated in an ongoing study (PROCARD 1b, NCT06832722). Given the stepwise increase in mortality above 30 ng/mL,^8^ using this threshold to initiate enzyme inhibition may enhance prognostic benefit, an approach that is currently under evaluation in this trial.

Whether similar associations between elevated cDPP3 and adverse outcomes exist in other forms of haemodynamic instability, such as shock secondary to acute decompensated heart failure (HF), remains to be established. Exploring cDPP3 in acute heart failure (AHF), a related but earlier stage of circulatory dysfunction in which patients are typically treated with RAAS inhibitors, may provide valuable insights into its pathophysiological role and prognostic utility before the onset of overt shock. The STRONG-HF and CORTAHF trials have each contributed important insights into the management of AHF and included cDPP3 measurements at baseline and follow-up.^9,10^ The present analysis of these trials aims to assess cDPP3 concentrations in AHF, determine the prognostic significance of cDPP3 concentrations ≥40 ng/mL at baseline in AHF, to explore how medical treatment may influence cDPP3 trajectory and related outcomes and to identify predictive factors associated with elevated concentrations at baseline, both at the prognostic cut-off of 40 ng/mL and the putative therapeutic threshold of 30 ng/mL.

## Methods

This work represents an analysis of two prospective studies, STRONG-HF and CORTAHF, which both enrolled patients hospitalized for AHF. Patients were included in the absence of identifiable and potentially reversible precipitants, resulting in two cohorts with comparable AHF trajectories, in which acute decompensation was considered to reflect intrinsic disease progression, based on clinical presentation rather than defined by a formal staging criterion. STRONG-HF was a multinational, multicentre, open-label, randomized, controlled trial designed to assess the safety and efficacy of rapid up-titration of guideline-directed medical therapy (GDMT) in patients hospitalized for AHF compared to usual care. The detailed study design has been previously published.^14^ In brief, 1085 patients hospitalized for AHF were enrolled and followed for 90 days with both biobank sampling and outcome assessment, and for 180 days for outcome assessment only. Patients randomized to the high-intensity care arm initiated immediate up-titration of renin–angiotensin system inhibitors (angiotensin converting-enzyme inhibitors [ACEIs], angiotensin receptor blockers [ARBs], or angiotensin receptor-neprilysin inhibitors [ARNIs]), beta-blockers, and mineralocorticoid receptor antagonists (MRAs), aiming to reach at least 50% of target doses before hospital discharge. Patients were then assessed at weeks 1, 2, 3 and 6, with clinical, standard laboratory measurements, and congestion evaluations performed at each timepoint. At the 2-week visit, full up-titration to optimal GDMT doses was targeted if deemed safe.

CORTAHF was a multicentre, open-label, randomized, controlled trial evaluating the short-term impact of corticosteroid therapy in patients with AHF.^15,16^ The study enrolled 100 patients, who were randomly assigned on day 1 to receive either oral prednisone 40 mg once daily for 7 days or standard AHF treatment. Patients were subsequently assessed on day 2, day 4 or at discharge if earlier, day 7, and day 30, with a final telephone follow-up at day 90.^16^ Blood samples for biobanking were collected at baseline, day 4 (or at discharge if earlier), day 7, and day 30. Both studies were approved by the relevant regulatory authorities, and ethical approval was obtained at all participating sites.

### cDPP3 measurement

In the STRONG-HF trial, blood samples for cDPP3 measurement were collected at baseline and at day 90 of follow-up. In the CORTAHF cohort, samples were obtained at baseline, day 4 (or at discharge if earlier), day 7, and day 30. Samples were drawn into tubes containing ethylenediaminetetraacetic acid, immediately frozen, and stored locally at −20°C or colder until shipment to a central depot, where they were maintained at −80°C. All samples were subsequently analysed for cDPP3 concentrations at 4TEEN4 Therapeutics GmbH (Hennigsdorf, Germany) using a luminescence immunoassay (4TEEN4 Pharmaceuticals GmbH, Berlin, Germany).^17^ For reference, in a general adult population of 8849 volunteers measured with this assay, the median cDPP3 concentration is 15 ng/mL, and the 97.5th percentile is 40 ng/mL.^8,9,18^

Measurement of cDPP3 in the two AHF cohorts showed that in the STRONG-HF cohort, 222 of 973 patients (22.8%) had cDPP3 levels greater than 40 ng/mL and 304 of 973 (31.2%) had levels greater than 30 ng/mL. In contrast, in the CORTAHF cohort, only 4 of 95 patients (4.2%) had cDPP3 levels greater than 40 ng/mL and 8 of 95 (8.4%) greater than 30 ng/mL. Because few patients in CORTAHF had cDPP3 concentrations above 40 ng/mL, outcome and treatment-response analyses were restricted to STRONG-HF, whereas predictors of elevated cDPP3 were explored using a pooled AHF dataset in an exploratory manner. Given the very low number of CORTAHF patients with cDPP3 ≥ 40 ng/mL, this analysis should be interpreted as hypothesis-generating and is primarily driven by the STRONG-HF cohort. Two cDPP3 cut-offs were considered to capture complementary aspects of risk: the 30 ng/mL threshold marks the transition above physiological levels and represents a potential therapeutic cut-off; the 40 ng/mL value corresponds to the 97.5th percentile of the general population, representing the upper limit of physiological concentrations, and is commonly used as a prognostic benchmark.

### Study objectives and endpoints

The primary objective of the study was to assess cDPP3 concentrations in AHF and the prognostic significance of baseline cDPP3 concentrations ≥40 ng/mL in AHF patients in relation to clinical outcomes, including all-cause mortality or HF readmission through 180 days in the STRONG-HF trial and death, worsening HF, or HF readmission through 90 days in the CORTAHF trial. Secondary objectives included the evaluation of how GDMT in STRONG-HF and medical treatment in CORTAHF influenced cDPP3 trajectories over time, up to day 90 in STRONG-HF and day 30 in CORTAHF, and the identification of clinical and biological factors associated with elevated cDPP3 concentrations at baseline, both at the prognostic cut-off of 40 ng/mL and the putative therapeutic threshold of 30 ng/mL.

### Statistical analysis

As *ex vivo* haemolysis can falsely elevate cDPP3, values from haemolysed samples were treated as missing in these analyses.

Differences in baseline characteristics between patients with and without elevated cDPP3 levels were examined in each study separately. Continuous variables were reported as mean ± standard deviation or median [interquartile range] for skewed variables and compared using the Student’s *t*-test or Wilcoxon rank-sum test, as appropriate. Categorical variables were summarized as counts and percentages and compared using the chi-square or Fisher’s exact test, while ordinal variables were compared using a test of non-zero correlation.

Comparisons of time-to-event outcomes by cDPP3 groups were analysed using Kaplan–Meier estimates and Cox proportional hazards models. As previously described for the STRONG-HF studies, analyses of 180-day clinical outcomes were restricted to patients enrolled at sites where patients were followed to 180 days, and down-weighted by half the number of patients who were enrolled before the change in the primary endpoint.

Changes in cDPP3 from baseline to day 90 in the STRONG-HF trial were analysed using ANCOVA models. Mean, standard deviation (SD), and median were calculated on non-transformed values. Geometric means (GM) and corresponding 95% confidence intervals (CIs) were back-transformed to the original scale. GM of change from baseline represents the ratio of the post-baseline value to the baseline value. Models were adjusted for treatment group (High-Intensity vs. Usual Care), baseline cDPP3, left ventricular ejection fraction (LVEF) category (≤40% vs. >40%), and geographic region. These covariates were included as potential confounders given their potential associations with outcomes: treatment group reflects management intensity, baseline cDPP3, disease severity, LVEF, and region healthcare and population differences. The same analysis was repeated by ethnicity. In the CORTAHF trial, longitudinal changes were evaluated using a mixed model for repeated measures (MMRM), including fixed effects for centre, treatment, visit, baseline log-cDPP3, and interaction terms (baseline-by-visit and treatment-by-visit).

To identify predictors of elevated cDPP3 concentrations (for both ≥40 ng/mL and ≥30 ng/mL), univariable and multivariable logistic regression analyses were performed. The final multivariable model was built using multiple imputed data and backward stepwise selection, with a significance threshold of *P* < .05. Analyses were performed on the combined available dataset (STRONG-HF and CORTAHF). Because STRONG-HF accounts for more than 90% of the pooled population, estimates from these models are largely driven by STRONG-HF.

A two-sided *P*-value <.05 was considered statistically significant. Statistical analyses were performed using SAS version 9.4 (SAS Institute Inc., Cary, NC, USA).

## Results

Haemolysed results were detected in 37/1010 (3.7%) baseline samples in STRONG-HF and 4/100 (4.0%) in CORTAHF; these patients were thus excluded from the analyses.

### Baseline population characteristics

In STRONG-HF, 222 (23%) patients had cDPP3 concentrations ≥40 ng/mL. The population characteristics of this cohort are summarized in *Table 1*. Patients with cDPP3 ≥ 40 ng/mL were statistically significantly younger (mean age 58 ± 15 vs. 65 ± 13 years, *P* < .0001), had a statistically significant higher prevalence of female sex (47.7% vs. 35.8%, *P* = .0013) and lower BMI (26.7 ± 6 vs. 28.8 ± 6 kg/m^2^, *P* < .0001). A statistically significant difference in ethnicity distribution was observed, with a greater proportion of Black patients in the higher cDPP3 group (42.8% vs. 14.4%, *P* < .0001). Regarding comorbidities, diabetes and atrial fibrillation were more prevalent in the low cDPP3 group (32.5% vs. 20.3% and 51.8% vs. 28.8%, respectively; both *P* < .001) as well as ischaemic aetiology of HF (52.5% vs. 36.5%); baseline LVEF was similar between groups (36%). The high cDPP3 group had statistically significantly higher eGFR (70 ± 25 vs. 63 ± 21 mL/min/1.73 m^2^, *P* < .0001) and a lower NT-proBNP (2724 pg/mL [95% CI 2523–2940] vs. 3339 pg/mL [95% CI 3191–3494]; *P* < .0001). This group was also more frequently treated with an MRA (99% vs. 94%, *P* = .001) and a loop diuretic (98% vs. 95%, *P* = .04).

**Table 1:** Baseline characteristics according to dipeptidyl peptidase 3 levels (cDPP3) in the STRONG-HF study

Parameter	Statistic	cDPP3 < 40 ng/mL (*N* = 751)	cDPP3 ≥40 ng/mL (*N* = 222)	*P*-value
Demographics				
Age, years	Mean (SD)	64.6 (12.64)	58.0 (14.74)	**<.0001**
Male sex	*n* (%)	482 (64.2%)	116 (52.3%)	.**0013**
Race				**<**.**0001**
Black	*n* (%)	108 (14.4%)	95 (42.8%)	
Caucasian or White	*n* (%)	631 (84.0%)	126 (56.8%)	
Pacific Islander	*n* (%)	0	1 (0.5%)	
Other	*n* (%)	12 (1.6%)	0	
BMI, kg/m^2^	Mean (SD)	28.8 (5.94)	26.7 (6.23)	**<**.**0001**
Medical history				
Diabetes	*n* (%)	243 (32.5%)	45 (20.3%)	.**0005**
Malignancies	*n* (%)	25 (3.3%)	3 (1.4%)	.1203
Moderate or severe COPD or asthma	*n* (%)	19 (2.5%)	4 (1.8%)	.5304
Stroke or transient ischaemic attack	*n* (%)	69 (9.2%)	22 (10.0%)	.7351
Atrial fibrillation or atrial flutter	*n* (%)	389 (51.8%)	64 (28.8%)	**<**.**0001**
Sustained ventricular arrhythmia	*n* (%)	1 (0.1%)	0	.5865
CRT	*n* (%)	3 (0.4%)	1 (0.5%)	.9169
ICD	*n* (%)	4 (0.5%)	2 (0.9%)	.5380
Heart failure history				
NYHA class				**<**.**0001**
Class I	*n* (%)	44 (6.3%)	8 (3.9%)	
Class II	*n* (%)	170 (24.3%)	103 (50.2%)	
Class III	*n* (%)	318 (45.5%)	60 (29.3%)	
Class IV	*n* (%)	167 (23.9%)	34 (16.6%)	
LVEF, %	Mean (SD)	36.4 (12.62)	35.6 (11.81)	.4357
Ischaemic aetiology	*n* (%)	393 (52.5%)	81 (36.5%)	**<**.**0001**
Baseline vital signs				
Systolic blood pressure at baseline, mmHg	Mean (SD)	122.5 (12.37)	124.7 (14.78)	.**0269**
Heart rate, bpm	Mean (SD)	77.7 (11.64)	82.1 (11.11)	**<**.**0001**
Laboratory findings				
Sodium, mmol/L	Mean (SD)	140.5 (4.11)	140.3 (4.03)	.5212
Potassium, mmol/L	Mean (SD)	4.3 (0.45)	4.2 (0.40)	.**0112**
Glucose, mmol/L	Mean (SD)	6.4 (2.47)	5.7 (1.58)	**<**.**0001**
AST, U/L	Mean (SD)	26.9 (14.79)	25.7 (17.16)	.3079
ALT, U/I	Mean (SD)	29.1 (39.89)	33.1 (58.29)	.2444
Total bilirubin, umol/L	Mean (SD)	17.8 (11.55)	16.0 (10.62)	.**0429**
Hemoglobin, g/L	Mean (SD)	136.3 (20.54)	136.6 (18.33)	.8000
Urea, mmol/L	Mean (SD)	8.1 (3.42)	7.4 (3.22)	.**0034**
Creatinine, umol/L	Mean (SD)	107.2 (28.85)	102.8 (25.28)	.**0447**
eGFR, mL/min/1.73m^2^	Mean (SD)	62.9 (20.82)	70.2 (24.91)	**<**.**0001**
White blood cells, 10^9^/L	Mean (SD)	7.0 (2.01)	6.8 (1.84)	.0715
Lymphocytes, %	Mean (SD)	26.8 (9.43)	30.0 (9.97)	**<**.**0001**
NT-proBNP, pg/mL	Geom. Mean (95% CI)	3339.3 (3191.0, 3494.4)	2724.2 (2523.5, 2940.9)	**<**.**0001**
HF therapy				
ACEi/ARBs/ARNI	*n* (%)	492 (65.6%)	142 (64.0%)	.6530
Beta-blockers	*n* (%)	264 (35.2%)	77 (34.7%)	.8876
MRA	*n* (%)	703 (93.7%)	220 (99.1%)	.**0013**
Loop diuretic	*n* (%)	712 (94.9%)	218 (98.2%)	.**0356**

NE, Not estimable; shown for comparisons with no variability or insufficient data.

ACEi, angiotensin-converting enzyme inhibitor; ALT, alanine aminotransferase; ARB, angiotensin receptor blocker; ARNI, angiotensin receptor–neprilysin inhibitor; AST, aspartate aminotransferase; BMI, body mass index; COPD, chronic obstructive pulmonary disease; CRT, cardiac resynchronization therapy; eGFR, estimated glomerular filtration rate; ICD, implantable cardioverter-defibrillator; LVEF, left ventricular ejection fraction; MRA, mineralocorticoid receptor antagonists; NT-proBNP, N-terminal pro-B-type natriuretic peptide; NYHA, New York Heart Association.

In the CORTAHF study, only 4 patients (4%) had cDPP3 concentrations ≥40 ng/mL, and baseline characteristics were similar between the two groups (Supplementary Table S1).

Given the observed difference in cDPP3 concentrations across ethnic groups, patients were further stratified according to self-reported ethnicity (Black vs. non-Black) to explore whether high cDPP3 levels identify a distinct clinical phenotype. Among patients with elevated cDPP3, Black individuals had statistically significant lower BMI (cDPP3 ≥ 40 ng/mL group: 22.2 ± 4 vs. 30 ± 6 kg/m^2^; cDPP3 < 40 ng/mL group: 24.8 ± 7 vs. 29.5 ± 6 kg/m^2^, interaction *P* = .001), lower total bilirubin and hepatic transaminases levels (cDPP3 ≥ 40 ng/mL group: ALT 17.8 ± 7.1 vs. 44.5 ± 75.1 U/L; cDPP3 < 40 ng/mL group: 27.1 ± 30.7 vs. 29.4 ± 41.2 U/L, interaction *P* = .001), as well as lower serum creatinine (cDPP3 ≥ 40 ng/mL group: 91.0 ± 19.9 vs. 111.7 ± 25.4 µmol/L; cDPP3 < 40 ng/mL group: 99.0 ± 43.7 vs. 108.5 ± 25.3 µmol/L interaction *P* = .018). NT-proBNP concentrations were also significantly lower in Black compared with non-Black patients (Supplementary Table S2).

### Outcomes

**Figure 1:**
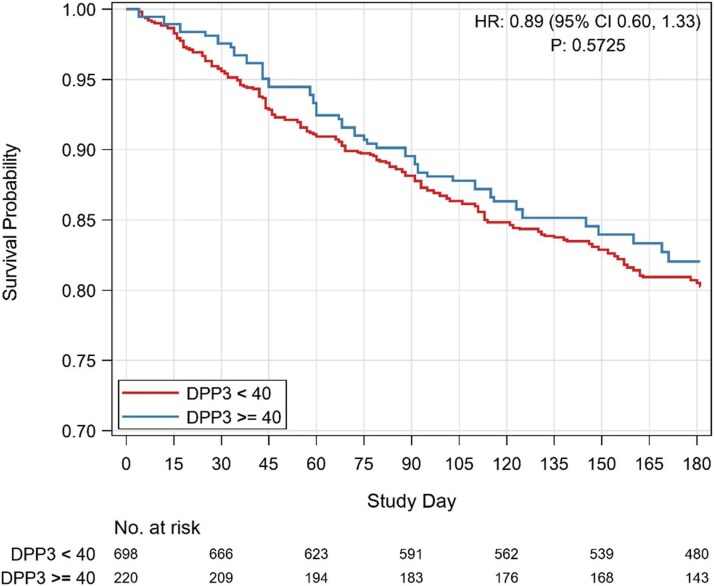
Clinical outcomes at 180 days according to baseline dipeptidyl peptidase 3 levels (cDPP3) in the STRONG-HF study. Kaplan–Meier curves showing event-free survival stratified by cDPP3 levels (<40 vs. ≥40 ng/mL). Hazard ratio (HR) and *P*-value refer to the comparison between groups. Numbers at risk are reported below the plot, and the *P*-value is .57. HR = hazard ratio; *P* = *P*-value; DPP3 = circulating dipeptidyl peptidase 3

Overall, no significant differences in clinical outcomes at 180 days were observed between patients with baseline cDPP3 < 40 ng/mL and those with DPP3 ≥ 40 ng/mL in either the STRONG-HF (*Figure 1*), nor at 90 days for the CORTAHF cohort (Supplementary Figure S1). In the STRONG-HF, however, Kaplan–Meier curves suggested a differential treatment effect: among patients with high cDPP3, those receiving High-Intensity Care experienced lower 180-day event rates compared with those with low cDPP3, whereas within the Usual Care group, high cDPP3 was associated with a higher risk of adverse outcomes compared with low cDPP3. This trend did not reach statistical significance (*P* for interaction = .30) (Supplementary Figure S2).

### Clinical outcomes by cDPP3 concentrations and ethnicity in the STRONG-HF cohort

To assess whether the prognostic value of cDPP3 concentrations varied by ethnicity, outcomes at 180 days were examined across cDPP3 categories (<40 vs. ≥40 ng/mL) within ethnic subgroups (Black vs. non-Black). No significant differences were observed between cDPP3 subgroups in either group (Supplementary Table S3).

### Change in cDPP3 concentrations over time according to treatment

In the STRONG-HF cohort, baseline median cDPP3 concentration were 20.7 ng/mL (range 4–650 ng/mL) in the High-Intensity Care group and 19.8 ng/mL (range 2–313 ng/mL) in the Usual Care group. Over time, adjusted geometric mean concentrations decreased to 0.85 of baseline in the High-Intensity Care group (−15% from baseline) and to 0.92 of baseline in the Usual Care group (−8% from baseline). In the mixed-effects model adjusted for baseline values, the ratio of geometric mean ratios (High-Intensity Care vs. Usual Care) was 0.922 (95% CI 0.842–1.009; *P* = .078), indicating a non-statistically significant difference (*Table 2*). To further assess whether cDPP3 changes paralleled improvements in congestion, we examined the association between change in NT-proBNP and change in cDPP3. NT-proBNP change was modelled both as a continuous log-transformed variable and using a clinically relevant responder definition (≥30% reduction from baseline to Day 90). After adjustment for baseline log(cDPP3), baseline log(NT-proBNP), LVEF category, region, and treatment group, neither continuous NT-proBNP change (*P* = .797) nor the ≥30% responder definition (*P* = .990) was associated with change in cDPP3. There was no evidence that these relationships differed by treatment strategy (treatment-by–NT-proBNP change interaction *P* = .404; treatment-by-responder interaction *P* = .545).

**Table 2:** Dipeptidyl peptidase 3 (cDPP3) trajectories from baseline to day 90 by treatment group in the STRONG-HF study

cDPP3 (ng/mL)	Statistic	High intensity care	Usual care	Total
Baseline	*n*	412	417	829
	Mean (SD)	36.0 (50.43)	35.6 (44.15)	35.8 (47.35)
	Median (Min, Max)	20.3 (4, 650)	19.1 (2, 313)	19.5 (2, 650)
	Geom. Mean	23.2	22.7	22.9
	95% CI	21.3, 25.2	20.9, 24.7	21.6, 24.3
Day 90	*n*	412	417	829
	Mean (SD)	30.3 (30.62)	36.0 (40.41)	33.2 (35.97)
	Median (Min, Max)	17.8 (4, 206)	18.6 (3, 306)	18.0 (3, 306)
	Geom. Mean	21.2	22.9	22.1
	95% CI	19.7, 23.0	21.0, 25.0	20.8, 23.4
Change from baseline to Day 90	*n*	412	417	829
	Mean (SD)	−5.7 (49.16)	0.4 (38.74)	−2.6 (44.30)
	Median (Min, Max)	−1.5 (−634, 159)	0.3 (−262, 183)	−0.7 (−634, 183)
	Geom. Mean	0.92	1.01	0.96
	95% CI	0.85, 0.99	0.94, 1.08	0.91, 1.01
LS Mean [a]		0.851	0.923	
LS Mean Difference [a]		0.922		
95% CI [a]		0.842, 1.009		
*P*-value [a]		0.0777		

SD, standard deviation; LS, least squares; CI, confidence interval.

In the CORTAHF study, baseline median cDPP3 concentrations were 14.5 ng/mL [IQR 11.3, 18.1 ng/mL]. No statistically significant differences between the two groups were observed by day 30 (Supplementary Table S4).

### Change in cDPP3 concentrations over time across racial groups: high-intensity vs. usual care in the STRONG-HF cohort

Given the observed differences in ethnic distribution at baseline, we examined whether the effect of High-Intensity Care on cDPP3 trajectories differed between Black and non-Black patients. Among Black individuals, adjusted geometric mean concentrations decreased to 0.94 of baseline in the High-Intensity Care group and to 1.03 of baseline in the Usual Care group. In the mixed-effects model adjusted for baseline values, the ratio of geometric mean ratios (High-Intensity Care vs. Usual Care) was 0.915 (95% CI 0.740–1.130; *P* = .41), indicating no statistically significant difference between treatment groups (Supplementary Table S5*A*).

Among non-Black patients, adjusted geometric mean concentrations decreased to 0.82 of baseline in the High-Intensity Care group and to 0.89 of baseline in the Usual Care group, with a ratio of geometric mean ratios of 0.919 (95% CI 0.831–1.016; *P* = .10) (Supplementary Table S5*B*).

The treatment-by-race interaction was not significant (*P* = .88), indicating that the effect of High-Intensity Care on cDPP3 reduction was consistent across ethnic groups.

### Predictors of high cDPP3 concentration in AHF

In an exploratory pooled analysis STRONG-HF and CORTAHF cohorts, multivariable analysis, performed using variables available in both studies, identified MRA use (OR 3.83; 95% CI: 1.47–9.96; *P* = .006) as an independent predictor of cDPP3 ≥ 40 ng/mL. In contrast, non-Black race (OR 0.43; 95% CI: 0.29–0.64; *P* < .0001), atrial fibrillation (OR 0.61; 95% CI: 0.42–0.88; *P* = .008), increasing NT-proBNP (OR 0.73 per doubling; 95% CI: 0.61–0.88; *P* = .0012), and higher NYHA class (OR 0.53; 95% CI: 0.36–0.78; *P* = .0014) were associated with lower odds of cDPP3 ≥ 40 ng/mL (*Table 3*). Given the potential influence of renal function on MRA prescription, we performed sensitivity analyses incorporating eGFR (MDRD formula) into the multivariable model. When eGFR was forced into the final model, it was not associated with cDPP3 ≥ 40 ng/mL (OR per 1 mL/min/1.73m^2^ 0.995; 95% CI 0.986–1.004; *P* = .242), and the association between MRA use and elevated cDPP3 remained unchanged (OR 4.22; 95% CI 1.55–11.50).

**Table 3:** Predictors of elevated dipeptidyl peptidase 3 levels (cDPP3 ≥ 40 ng/mL) in the combined STRONG-HF and CORTAHF study cohorts

		Univariable results		Multivariable results	
Parameter	OR for unit change of:	OR (95% CI)	*P*-value	OR (95% CI)	*P*-value
Age, years	1 year increase	0.97 (0.96, 0.98)	**<.0001**		
Sex	Male vs. Female	0.62 (0.46, 0.84)	.**0020**		
Non-Black Ethnicity	Yes vs. No	0.26 (0.19, 0.36)	**<**.**0001**	0.43 (0.29, 0.64)	**<**.**0001**
BMI, kg/m^2^	27.78 vs. 24.48	0.73 (0.65, 0.81)	**<**.**0001**		
	31.60 vs. 27.78	0.79 (0.71, 0.86)	NE		
Heart Rate, bpm	1 bpm increase	1.03 (1.02, 1.04)	**<**.**0001**		
Systolic blood pressure, mmHg	1 mmHg increase	1.01 (1.00, 1.02)	.1268		
LVEF, %	1% increase	0.98 (0.92, 1.04)	.4471		
Ischaemic aetiology	Yes vs. No	0.53 (0.39, 0.72)	**<**.**0001**		
Diabetes	Yes vs. No	0.55 (0.38, 0.78)	.**0009**		
COPD	Yes vs. No	0.72 (0.25, 2.05)	.5401		
Atrial fibrillation	Yes vs. No	0.39 (0.29, 0.54)	**<**.**0001**	0.61 (0.42, 0.88)	.**0082**
White blood cell count, 10^9^/L	1 × 109/L increase	0.93 (0.87, 1.01)	.0861		
Lymphocytes, %	1% increase	1.03 (1.02, 1.05)	**<**.**0001**		
Haemoglobin, g/L	1 g/L increase	1.00 (0.99, 1.01)	.7765		
Urea, mmol/L	1 mmol/L increase	0.92 (0.88, 0.97)	.**0026**		
Creatinine, umol/L	1 umol/L increase	0.97 (0.94, 1.00)	.**0418**		
Glucose, mmol/L	1 mmol/L increase	0.85 (0.78, 0.93)	.**0003**	0.93 (0.85, 1.00)	.0597
Sodium, mmol/L	1 mmol/L increase	0.99 (0.95, 1.03)	.5511		
Potassium, mmol/L	4.30 vs. 4.00	0.91 (0.81, 1.01)	.0738		
	4.60 vs. 4.30	0.78 (0.67, 0.91)	NE		
ALT, U/L	1 U/L increase	1.01 (0.99, 1.02)	.2688		
Total bilirubin, umol/L	1 umol/L increase	0.99 (0.97, 1.00)	.0541		
NT-proBNP, log2	Doubling	0.69 (0.57, 0.82)	**<**.**0001**	0.73 (0.61, 0.88)	.**0012**
JVP	≥6 cm vs <6 cm	0.28 (0.15, 0.52)	**<**.**0001**		
Rales	Present vs. No Rales	0.99 (0.64, 1.54)	.9584		
Edema	1+/2+/3+ vs. 0	0.12 (0.01, 2.14)	.1497		
NYHA Class	III/IV vs. I/II	0.35 (0.24, 0.51)	**<**.**0001**	0.53 (0.36, 0.78)	.**0014**
ACEi/ARBs/ARNI	Yes vs. No	0.96 (0.70, 1.31)	.7858		
MRA	Yes vs. No	3.50 (1.40, 8.75)	.**0075**	3.83 (1.47, 9.96)	.**0060**
Beta-blockers	Yes vs. No	1.02 (0.75, 1.39)	.9057		
Study	STRONG vs. CORTAHF	6.09 (2.32, 15.94)	.**0002**	1.33 (0.45, 3.94)	.6068

Results are based on univariate and multivariate logistic regression models. Odds ratios (OR) and 95% confidence intervals (CI) are reported.

ACEi, angiotensin-converting enzyme inhibitor; ALT, alanine aminotransferase; ARB, angiotensin receptor blocker; ARNI, angiotensin receptor–neprilysin inhibitor; BMI, body mass index; COPD, chronic obstructive pulmonary disease; JVP, jugular venous pressure; LVEF, left ventricular ejection fraction; MRA, mineralocorticoid receptor antagonist; NT-proBNP, N-terminal pro-B-type natriuretic peptide; NYHA, New York Heart Association.

When applying the 30 ng/mL cut-off, the direction of associations remained consistent, with MRA use associated with higher odds, whereas non-Black race and higher NT-proBNP concentrations being associated with lower odds of cDPP3 ≥ 30 ng/mL (Supplementary Table S6).

## Discussion

This is the first study evaluating cDPP3 concentrations in patients with AHF without overt haemodynamic instability. The first main result is that cDPP3 concentrations in this AHF population were only slightly higher than reference values reported in healthy individuals,^8^ supporting previous evidence that significant elevations may occur mainly in the presence of ongoing haemodynamic compromise.^3–5,9^

The second important observation is that baseline cDPP3 concentrations ≥40 ng/mL were not significantly associated with clinical outcomes in this AHF population. However, a closer examination of the Kaplan–Meier curves warrants attention: in STRONG-HF, patients with high cDPP3 concentrations who received High-Intensity Care showed the greatest survival benefit, whereas those in the Usual Care group exhibited lower survival rates. This pattern likely reflects the impact of optimized GDMT, which may have attenuated the adverse prognostic influence of elevated cDPP3, thereby masking its independent association with outcomes.

AHF, however, represents a complex stress condition characterized by neurohormonal dysregulation, with elevated levels of neurohormonal markers, including angiotensin II, together with congestion, hypoxia, cellular necrosis, endothelial dysfunction, increased inflammatory activation, and end-organ impairment.^19–22^ These processes, varying in severity, may act as potential triggers for the release of intracellular DPP3; nevertheless, the mechanisms governing this release, as well as its precise contribution to the acute evolution of the disease, remain incompletely understood. A transient increase, or an earlier dynamic change, in cDPP3 during the initial phase of decompensation could therefore be hypothesized and might help identify patients at risk of adverse outcomes, an aspect that may not have been captured within the mid-term timeframe of our analysis. Moreover, it should be acknowledged that HF aetiologies (including the distinction between *de novo* and acute-on-chronic presentations), along with different trigger factors, disease stages, and haemodynamic profiles, all influence outcomes and make the interpretation of these findings even more challenging.^23–25^

Considering that the longitudinal trajectory of cDPP3 has previously provided complementary prognostic insights, we investigated whether medical treatment was associated with changes in cDPP3 concentrations over time. Earlier studies focused on short-term dynamics, within hours and few days, where a decline in cDPP3 was associated with improved outcomes.^2–6,9^ In our analysis, a third key finding emerged: cDPP3 concentrations did not show any significant change over the mid-term follow-up (90 days in STRONG-HF), irrespective of treatment intensity. Moreover, despite widespread use of RAAS inhibitors, cDPP3 levels did not show modulation by these therapies, suggesting that its regulation may be independent of pharmacologic RAAS blockade. In addition, cDPP3 changes did not track with NT-proBNP reduction. Taken together, these findings may indicate either a lack of association between cDPP3 and processes of myocardial or vascular remodelling, or simply insufficient statistical power to detect subtle changes, and support the concept that cDPP3 is unlikely to represent a marker of congestion in AHF.

In the overall STRONG-HF population, we found that patients with cDPP3 > 40 ng/mL were more frequently younger, female, Black, and had a predominantly non-ischaemic aetiology, suggesting a potentially distinct clinical and biological phenotype; moreover, they exhibited better renal function, in contrast to earlier findings,^3,5,9^ which we interpret as likely reflecting more aggressive diuretic therapy in this group. According to existing literature, such a clinical phenotype may be consistent with hypertensive heart disease or peripartum cardiomyopathy, which are more commonly observed in younger Black women.^26^ In this context, we analysed predictors of elevated cDPP3 concentrations. Across both thresholds (≥30 and ≥40 ng/mL), higher risk of elevated cDPP3 was observed with MRA use and Black ethnicity, while markers of congestion were inversely related. These findings are consistent with the notion that cDPP3 is unlike to represent a marker of congestion. MRA use, instead, may reflect the underlying clinical phenotype, possibly hypertensive heart disease, as previously suggested, or represent a marker of greater baseline disease severity that justified prompt MRA initiation according to previous HF guidelines.^27–30^ This association was not explained by differences in renal function, as sensitivity analyses incorporating eGFR did not modify the results. Importantly, these associations were directionally consistent across both cut-offs, supporting the robustness of the observed pattern.

Finally, the observed ethnic differences justified a dedicated analysis of the Black subgroup to determine whether variations in cDPP3 translated into differences in treatment response or outcomes. However, the higher cDPP3 concentrations in this group did not correspond to worse outcomes or differential treatment effects, remaining a descriptive observation without clear prognostic implications. To date, there are no well-characterized DPP3 gene polymorphisms known to be enriched in individuals of African ancestry or robustly linked to cDPP3 concentrations. Moreover, currently available reference ranges and distributional data for cDPP3 have been derived predominantly from cohorts of European ancestry,^8^ and their generalizability across diverse ancestral backgrounds remains uncertain. These findings therefore highlight an important knowledge gap and underscore the need for future studies specifically designed to characterize cDPP3 biology and its prognostic relevance across different ethnic groups.

This study has several limitations that should be acknowledged. First, the pooled analysis combined data from two distinct clinical trials (STRONG-HF and CORTAHF), which differed in sample size, geographic representation, therapeutic interventions, and follow-up duration. The CORTAHF cohort, in particular, was too small to allow definitive conclusions, and its contribution to the pooled analyses was limited. Second, heterogeneity in terms of HF aetiology and clinical severity may limit the generalizability of certain findings, prevent a more granular clinical understanding, and complicate direct comparisons between cohorts. Third, detailed haemodynamic data were not available, restricting our ability to fully characterize the clinical profile associated with elevated cDPP3 levels. The absence of invasive or imaging-derived measures of perfusion and cardiac output limits mechanistic interpretation, particularly regarding cDPP3’s potential contribution to circulatory dysfunction. Fourth, although ethnic differences emerged in cDPP3 patterns, our ability to rigorously examine these differences was constrained by small sample sizes. As such, findings should therefore be considered exploratory and hypothesis-generating. Finally, the observational nature of this biomarker analysis within two clinical trials limits causal inference, and residual confounding cannot be excluded.

In conclusion, cDPP3 concentrations in AHF patients were only slightly higher than reference values, and baseline levels ≥40 ng/mL were not associated with adverse outcomes. cDPP3 values remained unchanged despite GDMT, suggesting that its regulation may be independent of pharmacologic RAAS blockade and unrelated to myocardial remodelling. Higher cDPP3 concentrations were not linked to congestion, but were independently associated with MRA use and Black ethnicity, possibly reflecting a distinct clinical phenotype. However, these ethnic differences did not translate into worse outcomes or treatment response.

cDPP3 may reflect a systemic stress response or cellular injury and could be a marker of short-term risk, a hypothesis that remains to be clarified. Further studies with more detailed clinical characterization, including aetiology, trigger factors, and haemodynamic data, are needed to determine whether cDPP3 can refine risk stratification and guide the intensity of AHF management. Of particular interest will be the evaluation of infection-associated acute decompensation, especially with respect to progression towards septic or mixed shock, a setting in which cDPP3 already has an established role as a marker of clinical severity and prognosis, and where its pathophysiological relevance needs to be further elucidated.

## Supplementary Material

xvag076_Supplementary_Data

## Declarations

### Data Availability

No data were generated or analysed for this manuscript.

### Pre-registered Clinical Trial Number

None supplied.
