# Supplementary material for: Circulating dipeptidyl peptidase 3 and outcomes in acute heart failure: an analysis of the STRONG-HF and CORTAHF studies
Source: ESC Heart Fail. 2026 Mar 16;13(3):xvag076. doi: 10.1093/eschf/xvag076 (PMC13187924; doi:10.1093/eschf/xvag076)
Supplement: xvag076_Supplementary_Data [file xvag076_supplementary_data.zip › Supplementary Material.docx]

**Supplementary Material**

**Supplementary Figures and Tables**

**Figure 1. Clinical Outcomes at 90 Days According to Baseline Dipeptidyl Peptidase 3 Levels (cDPP3) in the CORTAHF Study.**

**
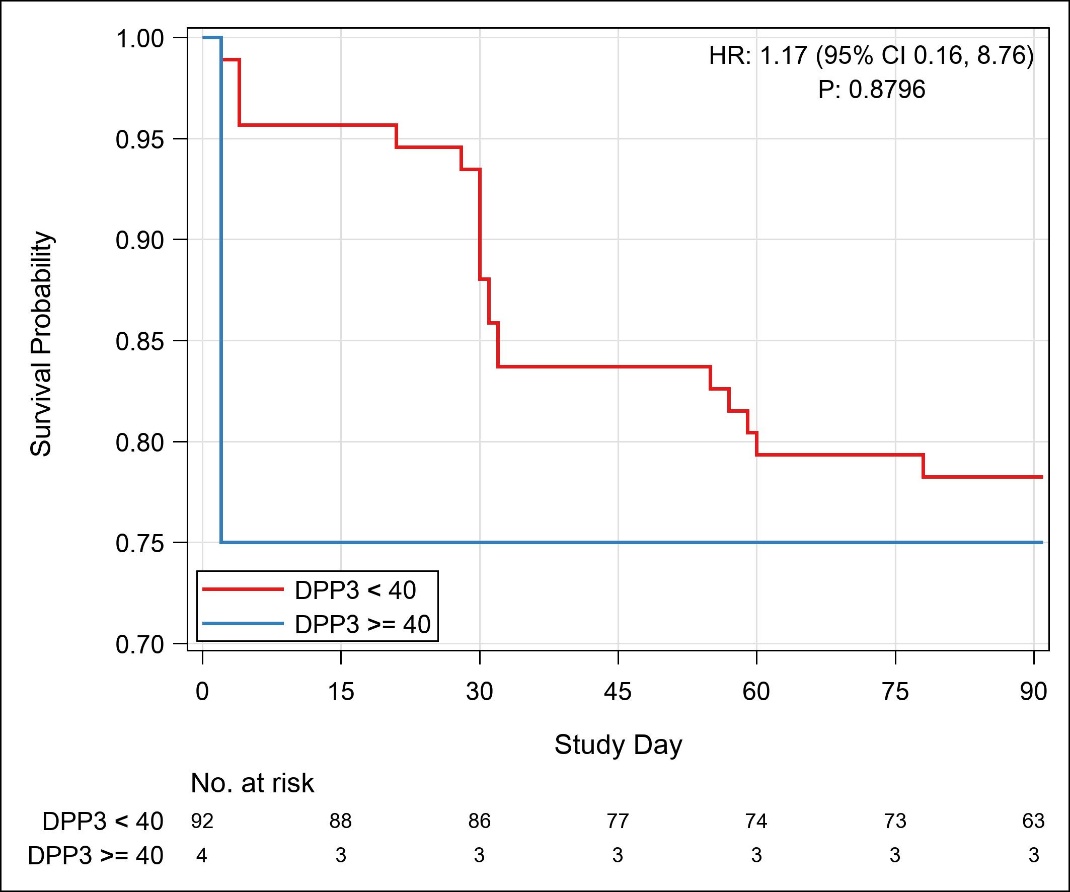
**

Kaplan–Meier curves showing event-free survival stratified by cDPP3 levels (<40 vs. ≥40 ng/mL). Hazard ratio (HR) and p-value refer to the comparison between groups.

Abbreviations: HR = hazard ratio; p = p-value; DPP3 = circulating dipeptidyl peptidase 3.

**Figure 2.**  **Clinical Outcomes at 180 Days According to Baseline Dipeptidyl Peptidase 3 Levels (cDPP3) and Treatment Strategy in the STRONG-HF trial.**

**
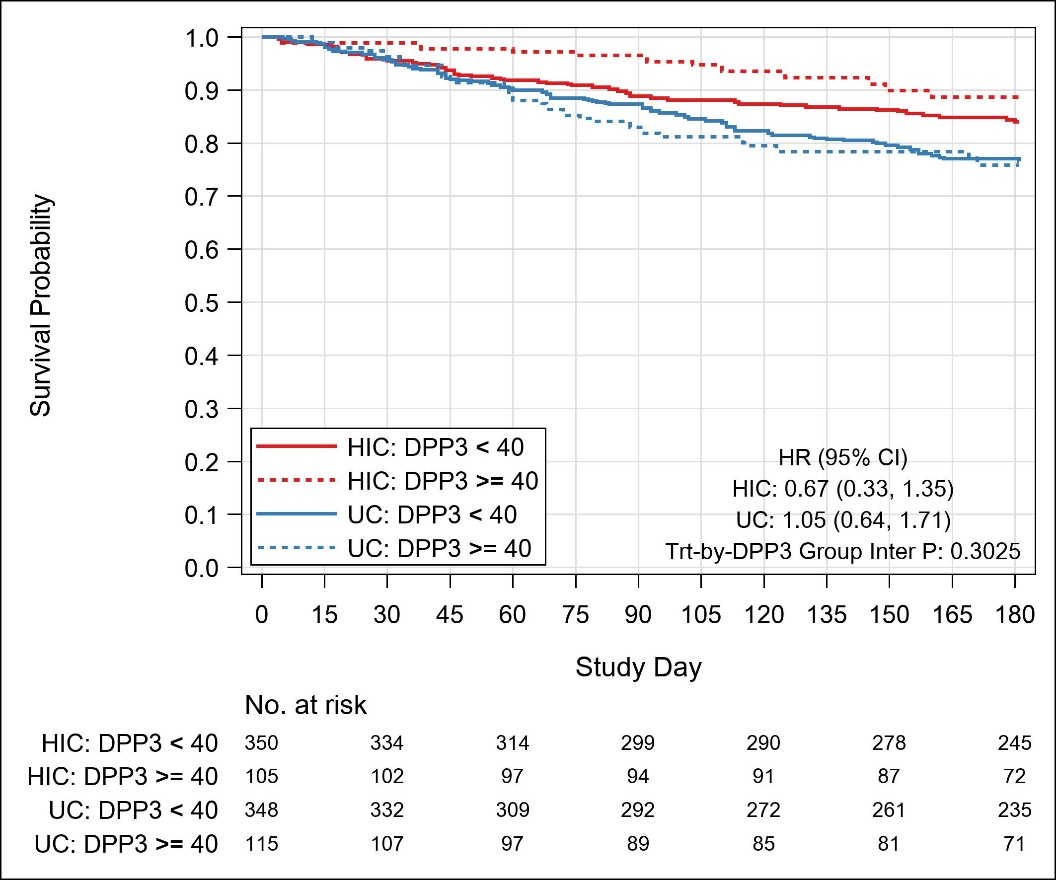
**

Kaplan–Meier curves showing event-free survival stratified by cDPP3 levels (<40 vs. ≥40 ng/mL). Hazard ratio (HR) and p-value refer to the comparison between groups.

Abbreviations: HR = hazard ratio; p = p-value; DPP3 = circulating dipeptidyl peptidase 3; HIC = high-intensity care; UC = usual care.

**Table 1.** Baseline Characteristics According to Dipeptidyl Peptidase 3 Levels (cDPP3) in the CORTAHF Study.

| Parameter | Statistic | cDPP3 <40 ng/mL (N= 92) | cDPP3 >=40 ng/mL (N= 4) | P-value |
| --- | --- | --- | --- | --- |
|  | | | | |
| Demographics |  |  |  |  |
| Age, years | Mean (SD) | 66.4 (8.90) | 68.5 (6.56) | 0.6449 |
| Male sex | n (%) | 59 (64.1%) | 3 (75.0%) | 0.6563 |
| Race |  |  |  |  |
| White | n (%) | 92 (100%) | 4 (100%) | NE |
| BMI, kg/m^2^ | Mean (SD) | 31.1 (5.69) | 29.9 (4.20) | 0.6887 |
| Medical history |  |  |  |  |
| Diabetes | n (%) | 39 (42.4%) | 2 (50.0%) | 0.7633 |
| Malignancy | n (%) | 2 (2.2%) | 0 | 0.7657 |
| COPD | n (%) | 8 (8.7%) | 0 | 0.5379 |
| Stroke or transient ischaemic attack | n (%) | 5 (5.4%) | 0 | 0.6320 |
| Atrial fibrillation or atrial flutter | n (%) | 23 (25.0%) | 2 (50.0%) | 0.2647 |
| Sustained ventricular arrhythmia | n (%) | 0 | 0 | NE |
| CRT | n (%) | 0 | 0 | NE |
| ICD | n (%) | 3 (3.3%) | 0 | 0.7137 |
| Heart failure history |  |  |  |  |
| NYHA class |  |  |  | 0.4644 |
| Class I | n (%) | 0 | 0 |  |
| Class II | n (%) | 15 (16.3%) | 0 |  |
| Class III | n (%) | 66 (71.7%) | 4 (100.0%) |  |
| Class IV | n (%) | 11 (12.0%) | 0 |  |
| LVEF, % | Mean (SD) | 28.4 (8.77) | 29.0 (7.79) | 0.8976 |
| Ischemic Aetiology | n (%) | 80 (87.0%) | 4 (100%) | 0.4400 |
| Baseline vital signs |  |  |  |  |
| Systolic blood pressure, mmHg | Mean (SD) | 142.9 (29.81) | 115.3 (8.96) | 0.0689 |
| Heart rate, bpm | Mean (SD) | 95.4 (11.12) | 97.0 (7.07) | 0.7771 |
| Laboratory findings |  |  |  |  |
| Sodium, mmol/L | Mean (SD) | 141.3 (3.32) | 142.0 (4.55) | 0.6977 |
| Potassium, mmol/L | Mean (SD) | 4.3 (0.57) | 4.6 (0.39) | 0.3629 |
| Glucose, mmol/L | Mean (SD) | 7.6 (3.18) | 8.1 (3.03) | 0.7430 |
| AST, U/L | Mean (SD) | 24.4 (14.74) | 18.5 (4.49) | 0.4299 |
| ALT, U/L | Mean (SD) | 28.3 (29.49) | 16.2 (6.66) | 0.4139 |
| Total bilirubin, umol/L | Mean (SD) | 13.5 (6.78) | 10.1 (7.46) | 0.3301 |
| Hemoglobin, g/L | Mean (SD) | 135.8 (20.01) | 138.3 (23.16) | 0.8145 |
| Urea, mmol/L | Mean (SD) | 8.4 (2.49) | 6.9 (0.30) | 0.2294 |
| Creatinine, umol/L | Mean (SD) | 106.9 (21.17) | 99.0 (6.38) | 0.4607 |
| eGFR, mL/min/1.73m^2^ | Mean (SD) | 60.7 (12.50) | 65.8 (7.46) | 0.4269 |
| White blood cells, 10^9^/L | Mean (SD) | 9.1 (2.92) | 9.2 (2.71) | 0.9715 |
| Lymphocytes, % | Mean (SD) | 20.8 (9.02) | 19.1 (10.41) | 0.7245 |
| NT-proBNP, pg/mL | Geom. Mean (95% CI) | 5205.0 (4331.6, 6254.4) | 4174.5 (1932.3, 9018.7) | 0.6235 |
| HF therapy |  |  |  |  |
| ACEi/ARBs/ARNI | n (%) | 62 (67.4%) | 4 (100.0%) | 0.1684 |
| Beta-blockers | n (%) | 56 (60.9%) | 4 (100.0%) | 0.1135 |
| MRA | n (%) | 34 (37.0%) | 1 (25.0%) | 0.6267 |
| Diuretic | n (%) | 57 (62.0%) | 3 (75.0%) | 0.5978 |

NE = Not estimable; shown for comparisons with no variability or insufficient data.

Abbreviations: ACEi = Angiotensin-converting enzyme inhibitor; ALT = Alanine aminotransferase; ARB = Angiotensin receptor blocker; ARNI = Angiotensin receptor–neprilysin inhibitor; AST = Aspartate aminotransferase; BMI = Body mass index; COPD = Chronic obstructive pulmonary disease; CRT = Cardiac resynchronization therapy; eGFR = Estimated glomerular filtration rate; ICD = Implantable cardioverter-defibrillator; LVEF = Left ventricular ejection fraction; MRA = Mineralocorticoid receptor antagonists; NT-proBNP = N-terminal pro-B-type natriuretic peptide; NYHA = New York Heart Association.

**Table 2.** Baseline Population Characteristics of STRONG-HF Stratified by Dipeptidyl Peptidase 3 Levels (cDPP3) and Race.

|  | | cDPP3 <40  ng/mL | | cDPP3 >=40 ng/mL | |  |
| --- | --- | --- | --- | --- | --- | --- |
| Parameter | Statistic | Not Black (N=643) | Black (N=108) | Not Black (N=127) | Black (N=95) | Interaction p-value |
| Demographics |  |  |  |  |  |  |
| Age, years | Mean (SD) | 66.7 (10.50) | 51.8 (16.30) | 65.4 (11.37) | 48.0 (12.77) | 0.2204 |
| Male sex | n (%) | 440 (68.4%) | 42 (38.9%) | 85 (66.9%) | 31 (32.6%) | 0.5703 |
| BMI, kg/m^2^ | Mean (SD) | 29.5 (5.56) | 24.8 (6.60) | 30.0 (5.51) | 22.2 (3.94) | **0.0010** |
| Medical History |  |  |  |  |  |  |
| Diabetes | n (%) | 236 (36.8%) | 7 (6.5%) | 40 (31.5%) | 5 (5.3%) | 0.9924 |
| Malignancies | n (%) | 25 (3.9%) | 0 | 3 (2.4%) | 0 | 0.8107 |
| Moderate or severe COPD or asthma | n (%) | 19 (3.0%) | 0 | 4 (3.1%) | 0 | 0.9897 |
| Stroke or transient ischaemic attack | n (%) | 68 (10.6%) | 1 (0.9%) | 17 (13.4%) | 5 (5.3%) | 0.1811 |
| Atrial fibrillation or atrial flutter | n (%) | 381 (59.3%) | 8 (7.4%) | 64 (50.4%) | 0 | 0.1021 |
| Sustained ventricular arrhythmia | n (%) | 1 (0.2%) | 0 | 0 | 0 | 0.8804 |
| CRT | n (%) | 3 (0.5%) | 0 | 1 (0.8%) | 0 | 0.7728 |
| ICD | n (%) | 4 (0.6%) | 0 | 2 (1.6%) | 0 | 0.6735 |
| Heart failure history |  |  |  |  |  |  |
| NYHA class |  |  |  |  |  | **0.0004** |
| Class I | n (%) | 20 (3.3%) | 24 (24.5%) | 4 (3.6%) | 4 (4.3%) |  |
| Class II | n (%) | 131 (21.8%) | 39 (39.8%) | 52 (46.4%) | 51 (54.8%) |  |
| Class III | n (%) | 284 (47.3%) | 34 (34.7%) | 22 (19.6%) | 38 (40.9%) |  |
| Class IV | n (%) | 166 (27.6%) | 1 (1.0%) | 34 (30.4%) | 0 |  |
| LVEF, % | Mean (SD) | 37.2 (12.67) | 31.5 (11.24) | 35.6 (12.15) | 35.7 (11.40) | **0.0069** |
| Baseline vital signs |  |  |  |  |  |  |
| Systolic blood pressure at baseline, mmHg | Mean (SD) | 121.4 (10.86) | 128.8 (17.84) | 121.5 (10.21) | 128.9 (18.52) | 0.9915 |
| Laboratory findings |  |  |  |  |  |  |
| Sodium, mmol/L | Mean (SD) | 141.0 (4.02) | 137.4 (3.24) | 142.1 (4.06) | 137.9 (2.39) | 0.2906 |
| Potassium, mmol/L | Mean (SD) | 4.3 (0.42) | 4.1 (0.49) | 4.4 (0.35) | 4.0 (0.34) | 0.0748 |
| Glucose, mmol/L | Mean (SD) | 6.6 (2.58) | 5.3 (1.29) | 6.3 (1.82) | 4.9 (0.58) | 0.7432 |
| AST, U/L | Mean (SD) | 26.9 (14.41) | 26.9 (16.98) | 31.1 (19.63) | 18.7 (9.41) | **<.0001** |
| ALT, U/I | Mean (SD) | 29.4 (41.24) | 27.1 (30.68) | 44.5 (75.06) | 17.8 (7.06) | **0.0014** |
| Total bilirubin, umol/L | Mean (SD) | 18.2 (11.23) | 15.1 (13.11) | 19.4 (12.79) | 11.4 (2.97) | **0.0126** |
| Hemoglobin, g/L | Mean (SD) | 138.5 (20.43) | 122.6 (15.33) | 142.0 (19.91) | 129.4 (12.93) | 0.3097 |
| Urea, mmol/L | Mean (SD) | 8.4 (3.39) | 6.4 (3.03) | 8.6 (3.66) | 5.8 (1.39) | 0.1856 |
| Creatinine, umol/L | Mean (SD) | 108.5 (25.31) | 99.0 (43.74) | 111.7 (25.36) | 91.0 (19.86) | **0.0180** |
| eGFR, mL/min/1.73m^2^ | Mean (SD) | 59.4 (17.08) | 83.7 (27.92) | 57.7 (16.71) | 87.1 (24.20) | 0.1233 |
| White blood cells, 10^9^/L | Mean (SD) | 7.2 (2.02) | 5.9 (1.56) | 7.4 (1.88) | 5.9 (1.45) | 0.7460 |
| Lymphocytes, % | Mean (SD) | 26.0 (8.80) | 31.8 (11.44) | 26.6 (8.99) | 34.4 (9.45) | 0.1915 |
| NT-proBNP, pg/mL | Geom. Mean (95% CI) | 3291.0 (3133.9, 3456.0) | 3641.4 (3221.6, 4115.8) | 3006.1 (2674.6, 3378.7) | 2388.2 (2199.5, 2593.0) | **0.0020** |
|  |  |  |  |  |  |  |
| HF therapy |  |  |  |  |  |  |
| ACEi/ARBs/ARNI | n (%) | 398 (61.9%) | 94 (87.9%) | 62 (48.8%) | 80 (84.2%) | 0.6134 |
| Beta-blockers | n (%) | 253 (39.3%) | 11 (10.3%) | 65 (51.2%) | 12 (12.6%) | 0.6095 |
| MRA | n (%) | 601 (93.5%) | 102 (95.3%) | 126 (99.2%) | 94 (98.9%) | 0.6662 |
| Loop diuretic | n (%) | 606 (94.2%) | 106 (99.1%) | 124 (97.6%) | 94 (98.9%) | 0.4988 |

Abbreviations: ACEi = Angiotensin-converting enzyme inhibitor; ALT = Alanine aminotransferase; ARB = Angiotensin receptor blocker; ARNI = Angiotensin receptor–neprilysin inhibitor; AST = Aspartate aminotransferase; BMI = Body mass index; COPD = Chronic obstructive pulmonary disease; CRT = Cardiac resynchronization therapy; eGFR = Estimated glomerular filtration rate; ICD = Implantable cardioverter-defibrillator; LVEF = Left ventricular ejection fraction; MRA = Mineralocorticoid receptor antagonists; NT-proBNP = N-terminal pro-B-type natriuretic peptide; NYHA = New York Heart Association.

**Table 3.** Clinical Outcomes by Dipeptidyl Peptidase 3 Levels (cDPP3) and Ethnicity in STRONG-HF.

|  |  | **cDPP3 at Baseline** | | **Unadjusted** | |
| --- | --- | --- | --- | --- | --- |
| **Outcome** | **Race Group** | **< 40 ng/mL**  **N=698** | **>= 40**  **ng/mL**  **N=220** | **HR/**  **LS-Mean Difference**  **(95% CI)**  **>=40 vs. < 40 ng/mL** | **Interaction**  **P-value** |
| **All-cause death or HF readmission by day 180** | Black | 19/104 (21.8%) | 18/95 (21.6%) | 0.99 (0.48, 2.02) | 0.5872 |
|  | Non- Black | 112/594 (19.5%) | 18/125 (15.2%) | 0.77 (0.44, 1.33) |  |

Abbreviations: HF = heart failure; CI = confidence interval.

**Table 4.** Dipeptidyl Peptidase 3 (cDPP3) Trajectories From Baseline to Day 30 by Treatment Group in the CORTAHF Study.

| Treatment Group | Statistic | Baseline | Day 4 or discharge | Day 7 | Day 30 |
| --- | --- | --- | --- | --- | --- |
|  | | | | | |
| Prednisone + Usual Care | n | 46 | 45 | 44 | 44 |
|  | Mean (SD) | 15.2 (5.99) | 14.2 (6.47) | 18.0 (18.24) | 17.3 (8.31) |
|  | Median (Q1, Q3) | 14.5 (11.3, 18.1) | 11.8 (10.2, 16.2) | 13.5 (9.2, 20.4) | 14.8 (11.3, 20.9) |
|  | Min, Max | 5, 29 | 6, 36 | 3, 113 | 8, 43 |
|  | Geom. Mean (SE) | 14.01 (0.86) | 13 (0.79) | 13.68 (1.44) | 15.77 (1.01) |
|  | | | | | |
|  | Change from Visit 1 |  |  |  |  |
|  | n |  | 43 | 42 | 42 |
|  | Mean (SD) |  | -1.0 (6.82) | 2.8 (17.71) | 1.9 (8.61) |
|  | Median (Q1, Q3) |  | -0.9 (-4.0, 2.1) | -1.6 (-5.4, 4.2) | 1.8 (-4.2, 5.2) |
|  | Min, Max |  | -17, 24 | -13, 92 | -16, 24 |
|  | Geom. Mean (SE) |  | 0.925 (0.055) | 0.967 (0.103) | 1.095 (0.077) |
|  |  |  |  |  |  |
|  | LS-Mean^*^ |  | 0.86 ( 1.06) | 0.91 ( 1.09) | 1.01 ( 1.07) |
|  | | | | | |
| Usual Care | n | 50 | 47 | 51 | 48 |
|  | Mean (SD) | 17.8 (12.67) | 17.2 (11.56) | 17.8 (11.69) | 14.8 (7.33) |
|  | Median (Q1, Q3) | 14.5 (10.5, 18.5) | 14.0 (10.8, 18.7) | 14.5 (11.4, 19.6) | 13.1 (9.2, 16.8) |
|  | Min, Max | 4, 63 | 4, 71 | 4, 69 | 5, 35 |
|  | Geom. Mean (SE) | 14.81 (1.21) | 14.72 (1.15) | 15.27 (1.16) | 13.21 (0.90) |
|  | | | | | |
|  | Change from Visit 1 |  |  |  |  |
|  | n |  | 45 | 49 | 47 |
|  | Mean (SD) |  | 0.7 (12.89) | 0.0 (14.63) | -3.5 (12.57) |
|  | Median (Q1, Q3) |  | 0.3 (-3.9, 5.6) | -0.1 (-2.9, 3.9) | -1.7 (-7.9, 3.5) |
|  | Min, Max |  | -27, 56 | -38, 38 | -39, 17 |
|  | Geom. Mean (SE) |  | 1.063 (0.083) | 1.027 (0.087) | 0.855 (0.070) |
|  |  |  |  |  |  |
|  | LS-Mean^*^ |  | 1.00 ( 1.06) | 0.99 ( 1.08) | 0.85 ( 1.06) |
|  | | | | | |
|  | LS Mean Difference (95% CI)^*^ |  | 0.86 ( 0.73, 1.01) | 0.92 ( 0.73, 1.16) | 1.20 ( 1.01, 1.43) |
|  | P-value^*^ |  | 0.0661 | 0.4613 | 0.0515 |
|  |  |  |  |  |  |
|  | Overall LS Mean Difference (95% CI) |  |  |  | 0.98 (0.86, 1.12) |
|  | Overall P-value |  |  |  | 0.7643 |
|  | | | | | |

Abbreviations: SD = standard deviation; LS = least squares; CI = confidence interval; SE = standard error; Q = interquartile range.

**Table 5a.** Dipeptidyl peptidase 3 (cDPP3) Trajectories From Baseline to Day 90 by Treatment Group in Black Patients in the STRONG-HF study.

| cDPP3 (ng/mL) | Statistic | High Intensity Care (N=115) | Usual  Care (N=115) | Total (N= 230) |
| --- | --- | --- | --- | --- |
|  | | | | |
| Baseline | n | 78 | 87 | 165 |
|  | Mean (SD) | 69.1 (91.02) | 54.3 (53.59) | 61.3 (73.83) |
|  | Median  (Min, Max) | 36.2 (5, 650) | 35.7 (7, 290) | 35.8 (5, 650) |
|  | Geom. Mean | 41.5 | 37.0 | 39.1 |
|  | 95% CI | 33.2, 51.8 | 30.8, 44.6 | 33.9, 45.0 |
|  | | | | |
| Day 90 | n | 78 | 87 | 165 |
|  | Mean (SD) | 49.1 (39.44) | 53.0 (50.24) | 51.1 (45.37) |
|  | Median  (Min, Max) | 33.7 (7, 164) | 33.5 (7, 306) | 33.5 (7, 306) |
|  | Geom. Mean | 35.3 | 35.5 | 35.4 |
|  | 95% CI | 29.2, 42.8 | 29.2, 43.1 | 30.9, 40.6 |
|  | | | | |
| Change from Baseline to Day 90 | n | 78 | 87 | 165 |
|  | Mean (SD) | -20.0 (90.92) | -1.3 (42.99) | -10.2 (70.27) |
|  | Median  (Min, Max) | -4.6 (-634, 126) | -0.3 (-210, 96) | -1.6 (-634, 126) |
|  | Geom. Mean | 0.85 | 0.96 | 0.91 |
|  | 95% CI | 0.70, 1.03 | 0.83, 1.11 | 0.81, 1.02 |
|  | | | | |
| LS Mean [a] |  | 0.941 | 1.028 |  |
| LS Mean Difference [a] |  | 0.915 |  |  |
| 95% CI [a] |  | 0.740, 1.130 |  |  |
| p-value [a] |  | 0.4066 |  |  |
|  | | | | |

[a] adjusted analyses.

Abbreviations: SD = standard deviation; LS = least squares; CI = confidence interval.

**Table 5b.** Dipeptidyl peptidase 3 (cDPP3) Trajectories From Baseline to Day 90 by Treatment Group in non-Black Patients in the STRONG-HF study.

| cDPP3 (ng/mL) | Statistic | High Intensity Care (N=427) | Usual  Care (N=419) | Total (N= 846) |
| --- | --- | --- | --- | --- |
|  | | | | |
| Baseline | n | 334 | 330 | 664 |
|  | Mean (SD) | 28.3 (30.14) | 30.7 (39.98) | 29.5 (35.36) |
|  | Median  (Min, Max) | 17.1 (4, 192) | 17.7 (2, 313) | 17.4 (2, 313) |
|  | Geom. Mean | 20.2 | 20.0 | 20.1 |
|  | 95% CI | 18.6, 21.9 | 18.2, 21.9 | 18.9, 21.3 |
|  | | | | |
| Day 90 | n | 334 | 330 | 664 |
|  | Mean (SD) | 26.0 (26.38) | 31.5 (36.18) | 28.7 (31.73) |
|  | Median  (Min, Max) | 15.6 (4, 206) | 16.5 (3, 221) | 16.0 (3, 221) |
|  | Geom. Mean | 18.9 | 20.4 | 19.6 |
|  | 95% CI | 17.4, 20.4 | 18.6, 22.4 | 18.4, 20.9 |
|  | | | | |
| Change from Baseline to Day 90 | n | 334 | 330 | 664 |
|  | Mean (SD) | -2.4 (31.81) | 0.8 (37.60) | -0.8 (34.81) |
|  | Median  (Min, Max) | -1.2 (-160, 159) | 0.3 (-262, 183) | -0.4 (-262, 183) |
|  | Geom. Mean | 0.93 | 1.02 | 0.98 |
|  | 95% CI | 0.86, 1.01 | 0.94, 1.11 | 0.92, 1.03 |
|  | | | | |
| LS Mean [a] |  | 0.820 | 0.892 |  |
| LS Mean Difference [a] |  | 0.919 |  |  |
| 95% CI [a] |  | 0.831, 1.016 |  |  |
| p-value [a] |  | 0.0990 |  |  |
|  |  |  |  |  |
| Interaction P-value[b] |  | 0.8770 |  |  |
|  | | | | |

[a] adjusted analyses.

Abbreviations: SD = standard deviation; LS = least squares; CI = confidence interval.

**Table 6.** Predictors of Elevated Dipeptidyl Peptidase 3 Levels (cDPP3 ≥30 ng/mL) in the Combined STRONG-HF and CORTAHF Study Cohorts.

|  | | Univariable Results | | Multivariable Results | |
| --- | --- | --- | --- | --- | --- |
| Parameter | OR for Unit Change of: | OR  (95% CI) | p-value | OR  (95% CI) | p-value |
| Age, years | 1 year increase | 0.96  (0.95, 0.97) | **<.0001** | 0.98  (0.97, 1.00) | **0.0065** |
| Sex | Male vs. Female | 0.68  (0.52, 0.89) | **0.0054** |  |  |
| Non-Black Race | Yes vs. No | 0.26  (0.19, 0.36) | **<.0001** | 0.38  (0.25, 0.56) | **<.0001** |
| BMI, kg/m2 | 27.78 vs. 24.48 | 0.73  (0.66, 0.81) | **<.0001** |  |  |
|  | 31.60 vs. 27.78 | 0.78  (0.72, 0.86) | **NE** |  |  |
| Heart Rate, bpm | 1 bpm increase | 1.03  (1.02, 1.04) | **<.0001** |  |  |
| Systolic blood pressure, mmHg | 1 mmHg increase | 1.01  (1.00, 1.02) | 0.1900 |  |  |
| LVEF, % | 1% increase | 0.97  (0.92, 1.03) | 0.3306 |  |  |
| Ischemic Aetiology | Yes vs. No | 0.55  (0.42, 0.72) | **<.0001** |  |  |
| Diabetes | Yes vs. No | 0.55  (0.40, 0.75) | **0.0002** |  |  |
| COPD | Yes vs. No | 0.54  (0.20, 1.46) | 0.2258 |  |  |
| Atrial Fibrillation | Yes vs. No | 0.47  (0.36, 0.63) | **<.0001** |  |  |
| White Blood Cell Count, 109/L | 1 ×109/L increase | 0.94  (0.88, 1.01) | 0.0709 |  |  |
| Lymphocytes, % | 1% increase | 1.03  (1.01, 1.04) | **0.0001** |  |  |
| Hemoglobin, g/L | 1 g/L increase | 1.00  (1.00, 1.01) | 0.4950 |  |  |
| Urea, mmol/L | 1 mmol/L increase | 0.92  (0.88, 0.97) | **0.0007** |  |  |
| Creatinine, umol/L | 1 umol/L increase | 0.97  (0.95, 1.00) | **0.0350** |  |  |
| Glucose, mmol/L | 1 mmol/L increase | 0.85  (0.79, 0.92) | **<.0001** | 0.92  (0.86, 0.99) | **0.0278** |
| Sodium, mmol/L | 1 mmol/L increase | 0.97  (0.94, 1.00) | 0.0721 |  |  |
| Potassium, mmol/L | 4.30 vs. 4.00 | 0.89  (0.80, 0.98) | **0.0089** | 1.00  (0.89, 1.12) | 0.0609 |
|  | 4.60 vs. 4.30 | 0.74  (0.65, 0.85) | NE | 0.82  (0.71, 0.94) | NE |
| ALT, U/L | 1 U/L increase | 1.01  (0.99, 1.02) | 0.3519 |  |  |
| Total bilirubin, umol/L | 1 umol/L increase | 0.99  (0.97, 1.00) | 0.0445 |  |  |
| NT-ProBNP, log2 | Doubling | 0.71  (0.61, 0.83) | **<.0001** | 0.73  (0.62, 0.85) | **0.0001** |
| JVP | >=6cm vs <6cm | 0.25  (0.15, 0.43) | **<.0001** |  |  |
| Rales | Present vs. No Rales | 0.91  (0.60, 1.36) | 0.6386 |  |  |
| Edema | 1+/2+/3+ vs. 0 | 0.55  (0.15, 2.04) | 0.3728 |  |  |
| NYHA Class | III/IV vs. I/II | 0.43  (0.31, 0.58) | **<.0001** |  |  |
| ACEi/ARBs/ARNI | Yes vs. No | 0.98  (0.74, 1.30) | 0.8981 |  |  |
| MRA | Yes vs. No | 3.37  (1.60, 7.11) | **0.0015** | 3.08  (1.40, 6.78) | **0.0053** |
| Beta-blockers | Yes vs. No | 1.05  (0.80, 1.39) | 0.7296 |  |  |
| Study | STRONG vs. CORTAHF | 5.00  (2.39, 10.44) | **<.0001** | 1.37  (0.59, 3.20) | 0.4671 |
