# Supplementary figures and images for: Circulating dipeptidyl peptidase 3 and outcomes in acute heart failure: an analysis of the STRONG-HF and CORTAHF studies
Source: ESC Heart Fail. 2026 Mar 16;13(3):xvag076. doi: 10.1093/eschf/xvag076 (PMC13187924; doi:10.1093/eschf/xvag076)

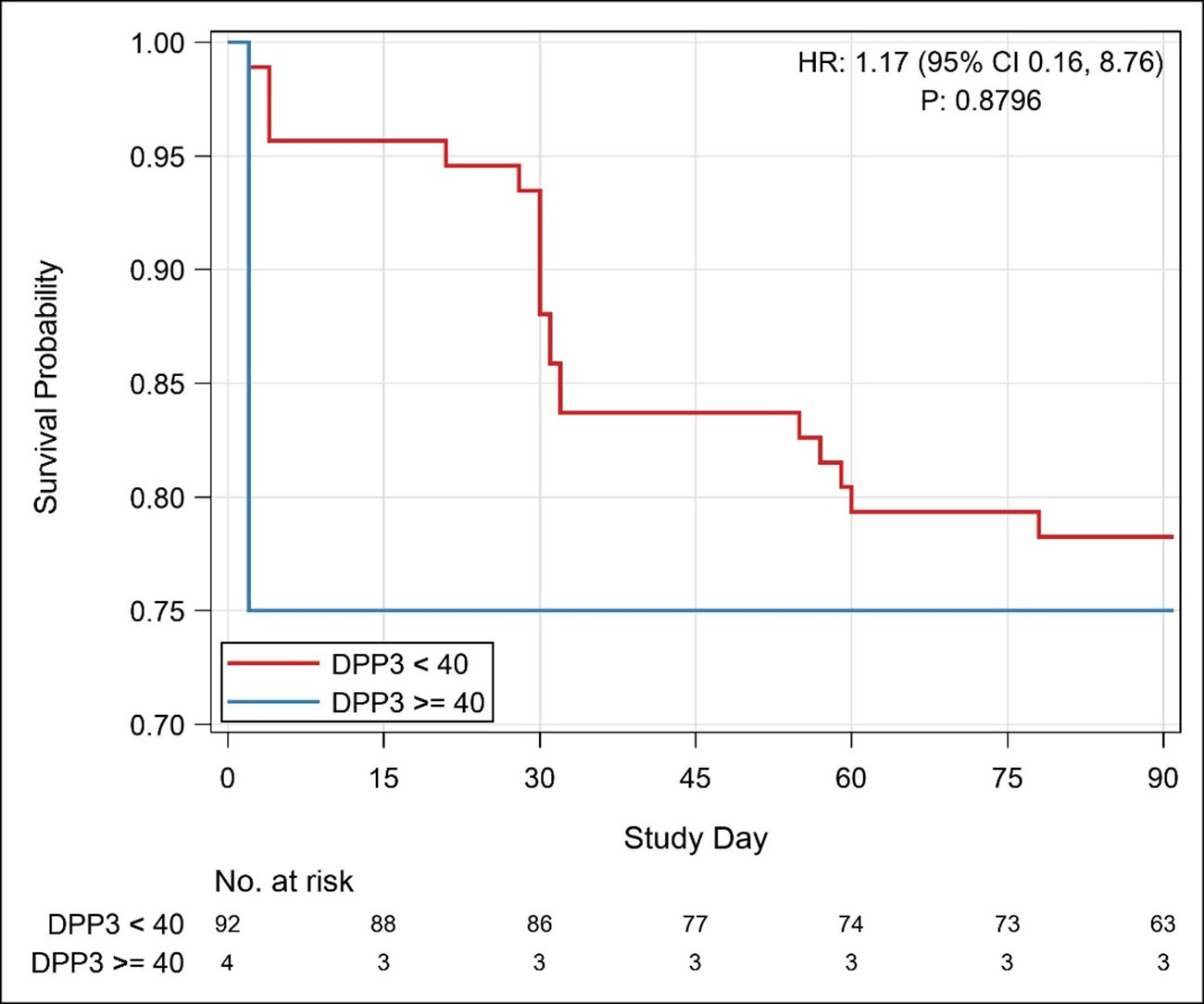

Supplement: xvag076_Supplementary_Data [file xvag076_supplementary_data.zip › Suppl Figure 1.jpg]

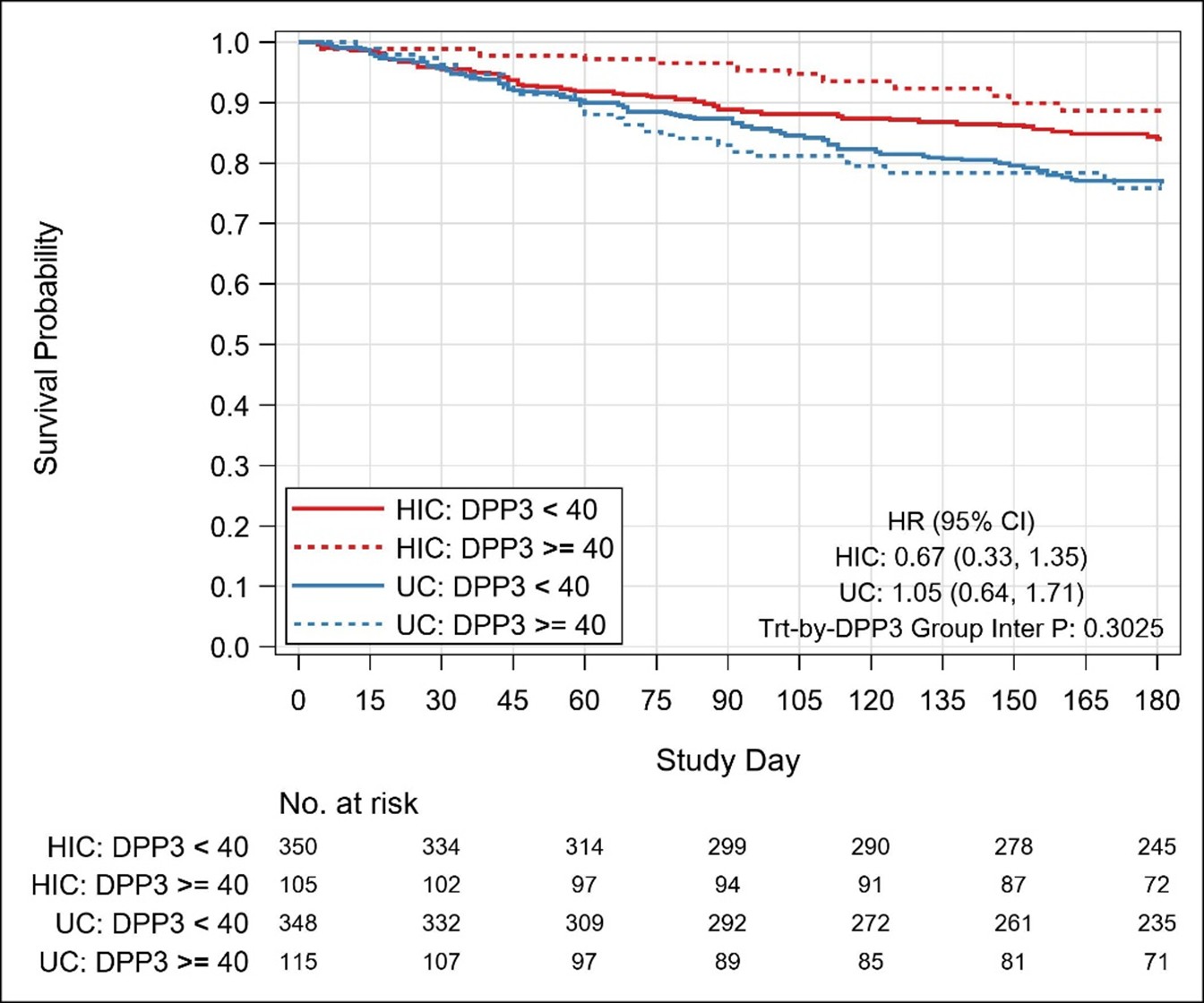

Supplement: xvag076_Supplementary_Data [file xvag076_supplementary_data.zip › Suppl Figure 2.jpg]
